# The Telomere Binding Protein Cdc13 and the Single-Stranded DNA Binding Protein RPA Protect Telomeric DNA from Resection by Exonucleases

**DOI:** 10.1016/j.jmb.2015.08.002

**Published:** 2015-09-25

**Authors:** Matthew Greetham, Emmanuel Skordalakes, David Lydall, Bernard A. Connolly

**Affiliations:** 1 Institute for Cell and Molecular Biology, The University of Newcastle, Newcastle upon Tyne NE2 4HH, United Kingdom; 2 The Wistar Institute, Philadelphia, PA 19104, USA

**Keywords:** DSB, double strand break, DBD, DNA binding domain, telomere, CST complex, Cdc13, RPA, exonuclease-catalyzed resection

## Abstract

The telomere is present at the ends of all eukaryotic chromosomes and usually consists of repetitive TG-rich DNA that terminates in a single-stranded 3′ TG extension and a 5′ CA-rich recessed strand. A biochemical assay that allows the *in vitro* observation of exonuclease-catalyzed degradation (resection) of telomeres has been developed. The approach uses an oligodeoxynucleotide that folds to a stem–loop with a TG-rich double-stranded region and a 3′ single-stranded extension, typical of telomeres. Cdc13, the major component of the telomere-specific CST complex, strongly protects the recessed strand from the 5′ → 3′ exonuclease activity of the model exonuclease from bacteriophage λ. The isolated DNA binding domain of Cdc13 is less effective at shielding telomeres. Protection is specific, not being observed in control DNA lacking the specific TG-rich telomere sequence. RPA, the eukaryotic single-stranded DNA binding protein, also inhibits telomere resection. However, this protein is non-specific, equally hindering the degradation of non-telomere controls.

## Highlights

• The free ends of telomeres need protection from nuclease-catalyzed degradation.
• Cdc13, one component of the telomere-specific CST complex, strongly protects telomeres.
• The isolated DNA binding domain of Cdc13 provides less protection than the entire protein.
• RPA, the single-stranded DNA binding protein, is also a powerful telomere shield.
• The work provides the first biochemical evidence that both Cdc13 and RPA are capable of protecting telomeres from exonuclease-catalyzed resection.

## Graphical abstract

**Figure:**
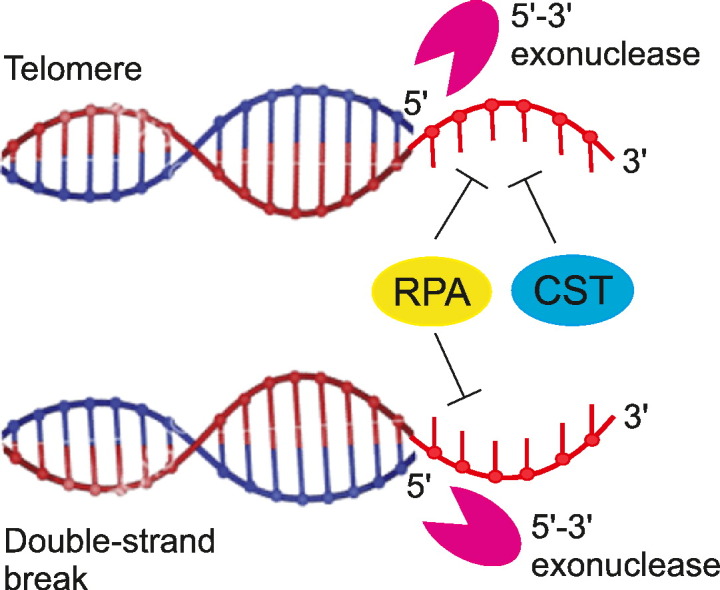

Telomeres occur at the ends of eukaryotic linear chromosomes. In *Saccharomyces cerevisiae*, telomeric DNA contains a repetitive TG_(1-3)_-rich double-stranded element, about 300 base pairs long, and terminates in a 3′ single-stranded overhang [1]. Telomeres resemble one-half of a double strand break (DSB), which can be formed by exposure to ionizing radiation or through replication fork collapse at unrepaired DNA lesions [2]. DSBs are actively repaired and, therefore, telomeres need to be hidden from the cellular DNA damage response machinery to prevent inappropriate checkpoint activation, end-to-end fusions and genome instability [3,4]. It remains unclear exactly how the structurally similar telomeres and DSBs are distinguished and, paradoxically, why so many of the proteins involved in DSB repair are also involved in telomere maintenance [5]. An obvious difference lies in the DNA sequence, with the TG_(1-3)_ repetitions being a telomere marker. The CST complex, a hetero-trimer assembled from Cdc13, Stn1 and Ten1, has been proposed to be a telomere-specific binding protein, with high affinity for the TG_(1-3)_ overhanging region [6,7]. Cdc13, the largest sub-unit of the CST complex, forms strong and specific complexes with telomere DNA sequences [7–10]. Much of the affinity arises from the action of the DNA binding domain (DBD) of Cdc13, a region of around 150 amino acids that contains an OB fold [7,11]. In yeast, the poor growth of cells expressing Cdc13-1 (a thermolabile version of Cdc13) at the non-permissive temperature can be rescued by deleting the 5′ → 3′ nuclease exonuclease Exo1 and other nuclease regulators [12]. Thus, one role of the CST complex may be to prevent telomere resection by nucleases such as Exo1 [9,12–17]. The CST complex has similarities to the replication protein RPA, the eukaryotic single-stranded DNA binding protein, and it has been suggested that CST is a telomere-specific RPA-like complex [18]. RPA is ubiquitous in eukaryotes and plays a key role in DNA metabolism by binding tightly to single-stranded DNA, preventing secondary structure formation and re-annealing during replication [19]. Both RPA and CST are essential hetero-trimeric proteins, which utilize OB folds to tightly bind single-stranded DNA [10,11,20]. Structural similarity is apparent between several of the component sub-units of the CST and RPA complexes [21,22], although a clear difference is the dimeric nature of Cdc13, a feature absent from RPA1 [23–27]. While CST is clearly a telomere-specific protein, chromatin immunoprecipitation analysis has indicated that RPA is also associated with telomeres and promotes telomerase action [28]. RPA plays a role in synthesis of the recessed strand through an interaction with DNA Polα [29]. Although the overlap in characteristics of RPA and CST makes the presence of RPA at telomeres unsurprising, the interplay between the two proteins remains uncertain, in particular, their individual roles in protecting chromosome ends from resection by exonucleases. This publication describes biochemical approaches toward elucidation of these questions.

To investigate the ability of Cdc13 and RPA to protect DNA ends from exonuclease-catalyzed degradation, we developed a protection assay using TL, a synthetic telomere analogue. As shown in Fig. 1, TL is a single oligodeoxynucleotide 90 bases in length, which forms a “snap-back” hairpin comprising two arms joined by a tetra-loop consisting of 4 thymidine bases. The folding of TL creates 34 base pairs of double-stranded DNA and a single-stranded 3′ extension, 16 bases in length. A fluorescein-labeled T derivative (X in Fig. 1) was located near the 3′ extremity, enabling monitoring of the binding of Cdc13 and RPA, as well as exonuclease-catalyzed resection; a phosphate group was present at the 5′ end. TL contains TG_(1-3)_ repeats, characteristic of *S*. *cerevisiae* telomeres, and a 16-base single-strand overhang that closely matches the 12- to 15-nucleotide extension seen through much of the cell cycle [1]. A non-telomere control (CL) of similar structure (Fig. 1) was derived from the Mata/Matα mating type locus in *S*. *cerevisiae*, which is a natural location for DNA DSB induction during mating-type switches [30]. The loop structure of both oligodeoxynucleotides is designed to model the fact that most telomeres and DSBs are likely to be several megabases away from the other end of the chromosome. The presence of only a single terminus in both TL and CL (a more typical double-stranded oligodeoxynucleotide would have two) ensures that exonucleolysis, either 3′ or 5′, can only commence at a single location, greatly simplifying analysis.

Genetic analysis in *S*. *cerevisiae* suggests that the CST complex may play a role in protecting the 5′ recessed strand of the telomere from resection by Exo1, a processive 5′ → 3′ exonuclease [12–17]. Purification of yeast Exo1 from Sf9 insect cells has been reported; however, miniscule amounts are produced, the level of homogeneity is low and the protein is unstable [31,32]. Our attempts to purify this enzyme were unsuccessful and, therefore, the exonuclease from bacteriophage λ (λ-exo), which has a powerful 5′ → 3′ exonuclease activity toward double-stranded DNA, high processivity and a strong preference for 5′-phosphates [33–35], has been used as a model nuclease. When TL and CL were treated with λ-exo, an intermediate of slightly greater mobility than the starting material was rapidly produced (Fig. 1). Comparison with standards identifies that the intermediate runs most closely to the marker that terminates at the 5′ T in the tetra-loop (standard C). Thus, the intermediate arises from exonucleolytic removal of the entire 5′ branch (34 bases) of the double-stranded region and predominantly consists of a single-strand of 56 bases, running from the first (5′) thymidine in the tetra-loop to the original 3′ terminus (Fig. 1). Given the gel resolution, it cannot be completely discounted that the intermediate also contains small amounts of slightly longer products, arising from instability and unwinding as the single-stranded region becomes very short. The intermediate was relatively stable and only more slowly converted into shorter, faster-running, fragments (Fig. 1). The properties of λ-exo [33–35] readily explain the persistence of this prominent intermediate; when the digestion reaches the 5′ loop thymidine, the DNA becomes single stranded, a poor substrate for the enzyme. Additionally, the high processivity of λ-exo accounts for the absence of bands between the starting material and intermediate product.

The ability of Cdc13 and RPA to inhibit degradation of the 5′ recessed strand of TL and CL by λ exonuclease was determined using 10 nM DNA and 50 nM protein. At these relative concentrations, electrophoretic mobility shift assays revealed that TL was completely bound by both Cdc13 and the isolated DBD of Cdc13; in contrast, absolutely interaction was seen with CL (data not shown). These observations agree with many previous studies, which show tight and specific binding of Cdc13 to telomeres [7–10]. In the absence of Cdc13, TL and CL were equally susceptible to hydrolysis by λ-exonuclease, with almost complete conversion of the starting material into the initial intermediate by the first time point of 10 s (Fig. 2). The intermediate persisted for about a minute, before degradation to smaller products, resulting from the slow activity of λ-exo on single-stranded DNA. When the reaction was carried out in the presence of Cdc13, resection was strongly inhibited with TL, as evidenced by the much slower conversion of the starting substrate to the intermediate, TL being clearly visible at the end of the digestion (270 s) (Fig. 2). Cdc13 also protects the intermediate product arising from TL digestion, no doubt as it remains bound to this single-stranded species. However, this secondary shielding is not of physiological relevance and arises solely as a consequence of the looped substrates used in these experiments. In contrast, Cdc13 offered no protection to CL and this material was degraded at the same rapid rate observed in the control when Cdc13 was omitted (Fig. 2). Parallel digestions were carried out using in the isolated DBD of Cdc13 to determine if this region is sufficient to provide protection from the λ-exonuclease catalyzed reaction. The gel patterns were reminiscent of those seen with the full-length protein in that absolutely no defense was afforded to CL by the DBD (Fig. 2). However, while DBD offered a degree of protection to TL, it was noticeably less potent than Cdc13 itself (Fig. 2). The gel illustrated in Fig. 2e provides some evidence for multiple products in the stable intermediate, probably due to premature termination by λ-exonuclease as the double-stranded region becomes very short and unstable. In a similar manner, any influence of RPA was investigated using 10 nM DNA and 50 nM protein. At these levels, electrophoretic mobility shift assay analysis indicated that both TL and CL were fully complexed by RPA (data not shown), in concurrence with earlier results showing strong, but non-specific, interaction with single-stranded DNA [36,37]. As expected, the two loops were rapidly degraded in the absence of added RPA; most of the initial substrate was destroyed after 20 s and the intermediate was prominent in both cases (Fig. 3). When RPA was added, very substantial protection was offered to both TL and CL with starting material clearly visible after 270 s, the last time point of the reaction (Fig. 3). Unusually, the stable intermediate was not observed when CL was digested with λ-exo in the presence of RPA for reasons that, at present, remain obscure. Overall, though, there appears little difference in the degree of protection that RPA affords the telomere and control loops.

To better compare the protective abilities of Cdc13 and RPA, we scanned the gels shown in Figs. 2 and 3 to determine the amount of substrate remaining throughout the time course, and we present these data in Fig. 4. In the case of Cdc13, inhibition of λ-exonuclease catalyzed resection is highly selective, with TL being strongly protected and CL not being shielded at all. Such profound discrimination is clearly a consequence of the specific binding of Cdc13 to telomere DNA sequences [6–10]. Although a role of Cdc13 in protecting telomeres from exonuclease resection *in vivo* has been demonstrated using genetic techniques [12–17], this is the first demonstration *in vitro* using biochemical approaches. The isolated DBD affords considerably less protection than full-length Cdc13 itself. It is likely that Cdc13 (924 amino acids), which is considerably larger than the single OB fold of around 200 amino acids that forms the DBD [7,10,11], provides a much greater degree of steric hindrance, explaining its enhanced potency. Further, there is evidence that the non-DBD OB folds in Cdc13 may be involved in DNA binding, as well as mediating protein–protein interactions [24,25]. The behavior of Cdc13 and RPA is starkly differentiated by substantial protection that RPA provides to the control CL loop, a substrate for which Cdc13 offers no defense against λ-exonuclease. Further, RPA appears to confer considerably more resistance to TL than the isolated DBD of Cdc13 and even protects the telomere to a greater extent than Cdc13 itself (Figs. 2–4). As RPA is able to bind all single-stranded DNA sequences, with minimal selectivity [36,37], the observed protection of both TL and CL is unsurprising.

In summary, the exonuclease assay developed in this publication, using hairpin oligodeoxynucleotides (which limit digestion to one end of the molecule) as model telomeres, has proved suitable for investigating resection by *in vitro* biochemical approaches. All experiments were carried out with the enzyme from bacteriophage λ (in these experiments, acting as a “general” exonuclease, that is, one able to degrade DNA but unlikely to be involved in additional protein–protein interactions with yeast proteins). Clearly, both Cdc13 and RPA are capable of shielding telomeres from destruction by “general” exonucleases, with RPA being somewhat more potent than Cdc13. It would be informative to repeat these experiments using more relevant nucleases from *S*. *cerevisiae* such as Exo1, unfortunately not available for these experiments due to purification difficulties [31,32]. Such investigations may reveal deviations from the simple protective default, mediated by protein–protein interactions between host yeast nucleases and CST or RPA. In this vein, it has been demonstrated that RPA stimulates the activity of Exo1 in resecting double-strand DNA breaks [30,39]. Finally, extending investigations from Cdc13 to the entire CST complex consisting of Cdc13–Stn1–Ten1 would be revealing. CST may be anticipated to offer more protection than Cdc13 alone, perhaps up to the high levels observed with RPA. An obvious candidate is the CST from *Candida glabrata* that can be purified and has been recently used to investigate telomere replication [40,41].

## Figures and Tables

**Fig. 1:**
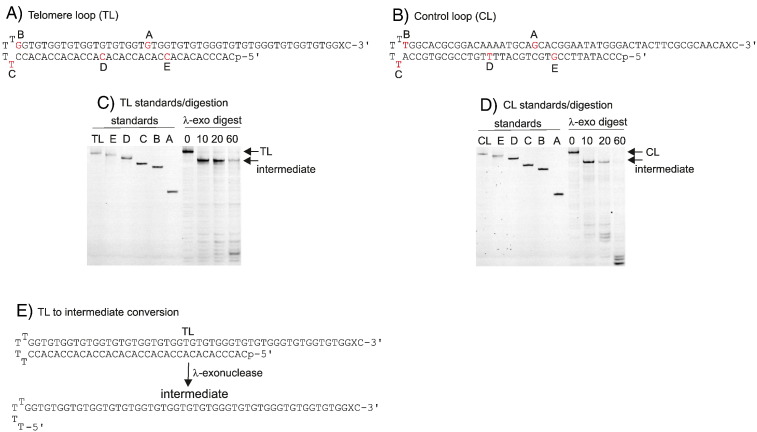
(A and B) The TG-rich, 90-base stem–loop (telomere loop, TL) used as a yeast telomere mimic and the stem–loop (control loop, CL) used as a control. Both TL and CL contain a 5′-phosphate (p) and X = fluorescein-dT. The oligodeoxynucleotides were obtained from ATDBio Ltd (Southampton, UK) and were supplied, HPLC purified and desalted. The 5′-phosphate was added using polynucleotide kinase and ATP (Promega) according to the supplier's instructions. After 5′-phosphorylation, TL and CL were purified from the enzyme and ATP using a PCR purification kit (Qiagen) and “folded” by heating to 90 °C in 20 mM Hepes, pH 7.5, 100 mM NaCl and 1 mM EDTA (*e*thylene*d*iamine*t*etraacetic *a*cid), followed by slow cooling to room temperature. A set of truncated standards that, commencing from the 3′ end, terminate at a base shown in red and are identified by letter were also purchased from ATDBio Ltd. These standards were not 5′-phosphorylated. (C and D) Digestion of TL and CL with λ-exonuclease followed by gel electrophoresis alongside the truncated standards. Digests were carried out at 37 °C using 10 nM of TL or CL in 67 mM glycine-KOH, pH 9.4, 2.5 mM MgCl_2_ and 0.01% (v/v) Triton X-100 (volume = 100 μl) with 50 units of λ-exonuclease (Fermentas/Thermo Fisher). Reactions were terminated, at the times indicated above the gels, by adding 20 μl of the reaction mixture to 20 μl of stop solution (90% formamide, 10 mM EDTA and 10 mM NaOH). Samples (20 μl) were then loaded onto a 17% denaturing (8 M urea) polyacrylamide gel run in Tris–borate–EDTA for 3 h at 3 W. Gels were imaged using a Typhoon FLA 9500 (GE) and analyzed with ImageQuant™ software. The starting TL/CL and the major intermediate are indicated by arrows. In both cases, the stable intermediate runs most closely to standard C and thus corresponds to the product formed by digestion up to the first (5′) T in the T loop. (E) Summary of the λ-exonuclease digestion with the structures of TL and the major intermediate. CL behaves identically. As stated in the text, the major intermediate may also contain traces of slightly longer product due to instability and unwinding of very short double-stranded regions produced as λ-exonuclease approaches the tetra-loop.

**Fig. 2:**
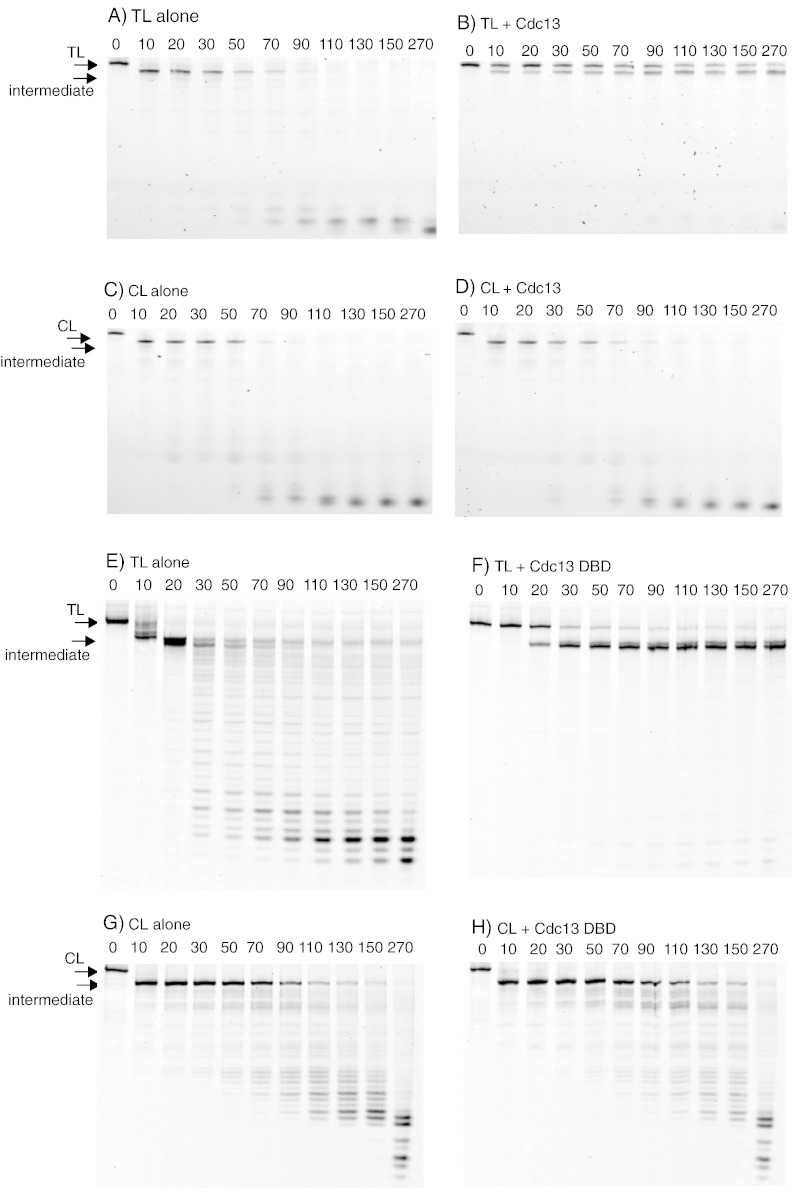
(A–H) Digestion of TL/CL by λ-exonuclease in the presence/absence of Cdc13 and Cdc13 DBD (DNA and protein combinations indicated above the gels). In all cases, the figures above the gel lanes represent the time of digestion in seconds. The positions of the starting TL and CL along with the stable intermediate are indicated with arrows. The digestions were carried out and analyzed exactly as described in Fig. 1. When Cdc13 and Cdc13 DBD were added, these were present at 50 nM. The two proteins were purified from *Escherichia coli* overexpressing strains. Cdc13 isolation used pET28b, which added a cleavable hexahistidine tag, removed post-purification with tobacco etch virus protease [25]. The DBD of Cdc13 (Cdc13-DBD) was purified as previously described, making use of pET21a and a C-terminal hexahistidine tag [8].

**Fig. 3:**
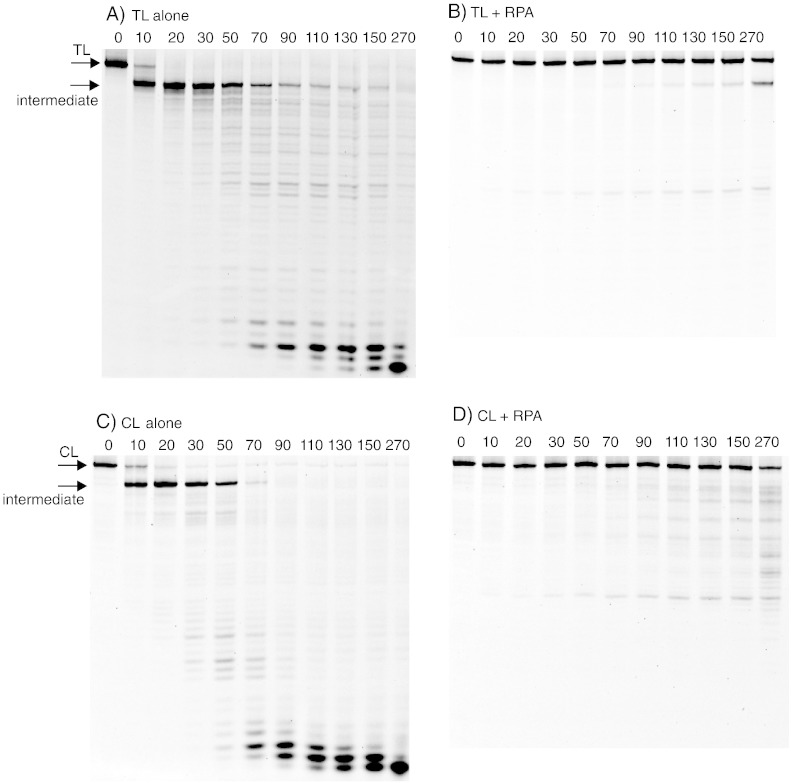
(A–D) Digestion of TL/CL by λ-exonuclease in the presence/absence of RPA (DNA and protein combinations indicated above the gels). In all cases, the figures above the gel lanes represent the time of digestion in seconds. The positions of the starting TL and CL along with the stable intermediate are indicated with arrows. The digestions were carried out and analyzed exactly as described in Fig. 1. When RPA was added, it was present at 50 nM. RPA was prepared as outlined previously using p11d-tRPA, a synthetic operon based on pET11 [38]. This system enables co-expression and co-purification of all three RPA sub-units (70, 32 and 14 kDa) as a complex and this species was used exclusively in this publication.

**Fig. 4:**
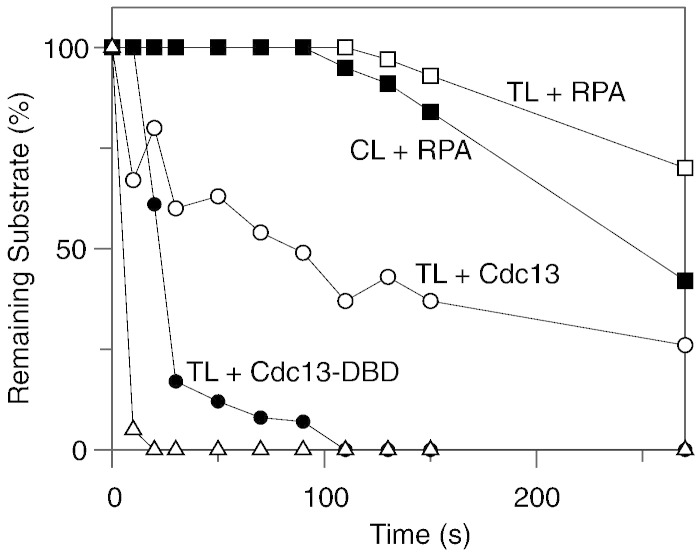
Comparison of the protection afforded to telomeres by Cdc13 and RPA from attack by exonuclease λ. Data were generated by scanning the gels shown in Figs. 2 and 3 and show the amount of starting TL or CL remaining at various digestion times. As illustrated, protection was observed with TL in the presence of Cdc13, Cdc13-DBD and RPA and with CL only with RPA. The unlabeled line with triangular data points typifies the digestion of free TL and CL and also of CL when Cdc13 was added; all were degraded at a very similar rapid rate and, hence, can be adequately represented with the single line show.
